# Regulation of gingival epithelial cytokine response by bacterial cyclic dinucleotides

**DOI:** 10.1080/20002297.2018.1538927

**Published:** 2018-11-27

**Authors:** Samira Elmanfi, Jie Zhou, Herman O. Sintim, Eija Könönen, Mervi Gürsoy, Ulvi Kahraman Gürsoy

**Affiliations:** a Department of Periodontology, Institute of Dentistry, University of Turku, Turku, Finland; b Department of Chemistry and Purdue Institute for Drug Discovery and Purdue Institute of Inflammation, Immunology and Infectious Disease, Purdue University, West Lafayette, IN, USA; c Oral Health Care, Welfare Division, City of Turku, Turku, Finland

**Keywords:** Lipopolysaccharide, inflammation, periodontitis, interleukin, keratinocytes

## Abstract

Background: Cyclic dinucleotides (cyclic di-guanosine monophosphate (c-di-GMP) and cyclic di-adenosine monophosphate (c-di-AMP)) and lipopolysaccharides (LPS) are pathogen-associated molecular patterns (PAMPs). Individual impacts of PAMPs on immune system have been evaluated, but simultaneous actions of multiple PAMPs have not been studied. Objective: Examination the effects of cyclic dinucleotides and *Porphyromonas gingivalis* LPS on gingival epithelial cytokine response. Methods: Human gingival keratinocytes (HMK) were incubated with 1, 10, and 100 µM concentrations of c-di-GMP and c-di-AMP, either in the presence or absence of *P. gingivalis* LPS. Intra- and extracellular levels of interleukin (IL)-1β, IL-8, IL-1Ra, monocyte chemoattractant protein (MCP)-1, and vascular endothelial growth factor (VEGF), were measured using the Luminex technique. Results: LPS decreased extracellular IL-8 levels, while the presence of c-di-AMP inhibited this effect. Incubating HMK cells with c-di-AMP (alone or with LPS) elevated the extracellular level of MCP-1. Extracellular VEGF level increased when cells were incubated with LPS and c-di-GMP together, or with c-di-AMP alone. LPS and c-di-AMP suppressed intracellular IL-1β levels. The c-di-AMP elevated intracellular levels of IL-1Ra. Conclusion: c-di-AMP and, to a lesser extent, c-di-GMP regulate keratinocyte cytokine response, either as an aggregator or as a suppressor of LPS, depending on the cytokine type.

## Introduction

Cyclic dinucleotides are important secondary signaling molecules in bacteria [1]. In Gram-negative bacteria, cyclic di-guanosine monophosphate (c-di-GMP) is involved in central bacterial processes, such as virulence, stress survival, motility, metabolism, biofilm formation, and differentiation [2]. Cyclic di-adenosine monophosphate (c-di-AMP) has a role in cell wall metabolism, DNA repair, and the control of gene expression in Gram-positive bacteria [3]. Cyclic dinucleotides contain either two guanine or two adenine bases linked to ribose and phosphate groups, and their synthesis is controlled by di-guanylate cyclases (DGCs) or di-adenosine cyclases (DACs) [4,5].

Information on cyclic dinucleotides in periodontal disease pathogenesis is limited and the presence and regulatory functions of the molecules in oral bacteria remain to be fully characterized. The presence of c-di-GMP, or its binding proteins, has been demonstrated in some periodontitis-associated bacteria, including *Porphyromonas gingivalis, Treponema denticola*, and *Selenomonas noxia* [6–9]. In addition, these secondary signaling molecules have been found to regulate the oxidative response, extracellular polysaccharide matrix production, and biofilm formation of cariogenic *Streptococcus mutans* [10,11].

Besides their role in intracellular signaling pathways, cyclic dinucleotides can trigger the innate immune response in mammalian cells [12]. Both bacterial-derived c-di-AMP and c-di-GMP, and host-derived cyclic guanosine monophosphate–adenosine monophosphate (cGAMP) bind to the stimulator of interferon genes (STING). Although the activation of the STING pathway and production of cytokines by cyclic dinucleotides in leukocytes are known, the effects of cyclic dinucleotides on other types of host cells remain poorly characterized. In periodontal tissues, various pathogen-associated molecular patterns (PAMPs), including lipopolysaccharides (LPS) and cyclic dinucleotides, are in contact with the gingival epithelium [13–15]. Activation of innate and adaptive immunity through alteration of gene expression by immune cells is mediated by Toll-like receptors (TLRs) that detect different classes of microorganisms or their by-products [16,17]. Upon recognition of bacteria and their by-products by cognate receptors, subfamilies of mitogen-activated protein kinases (MAPKs), namely extracellular signal–regulated kinases (ERKs), c-Jun amino-terminal kinases (JNKs), and p38 kinases, become activated. This eventually regulates the synthesis of proinﬂammatory cytokines and chemokines [18–20]. Of these, interleukin (IL)-1β is involved in many important cellular functions, such as proliferation, activation, and differentiation [21], whereas IL-1Ra blocks the activity of IL-1α and IL-1β by competition on their receptor binding [22]. Oral bacteria stimulate IL-8 and monocyte chemoattractant protein (MCP-1), which have the capacity to attract and activate neutrophils and monocytes, respectively, to the site of stimuli [23]. In addition, they act as mediators in the inflammatory response against pathogens [23]. Vascular endothelial growth factor (VEGF) plays an important role in wound healing and acts as a strong mitogen for endothelial cells [24].

*P. gingivalis*, a key periodontal pathogen, stimulates innate immune response and induces expression of inflammatory mediators; however, at the same time it downregulates selected host response mechanisms by disrupting their signaling pathways [13,25]. One of the most important virulence factors of *P. gingivalis* is its LPS, a constituent of the outer membrane, which is involved in the host-pathogen recognition and intracellular signaling events [14,26,27]. The transition to a dysbiotic bacterial community composition by *P. gingivalis* breaks the homeostatic relationship between the commensal microbiota and the host [14,27]. The shift from the dominance of Gram-positive bacteria to Gram-negative bacteria leads to changes in bacterial signaling molecule levels as well [28].

According to our hypothesis, cyclic dinucleotide levels have an impact on the cytokine response of human gingival keratinocytes. While effects of LPS on epithelial cells have been well characterized, those of cyclic dinucleotides remain to be elucidated. Due to the close contact between epithelial cells and various PAMPs simultaneously, it is likely that the overall response to pathogens is the aggregate of various stimuli. Therefore, the aims were to examine the effects of c-di-GMP and c-di-AMP on epithelial cytokine production and the activation of MAPKs in the presence and absence of *P. gingivalis* LPS.

## Material and methods

### Cell culture

Human gingival keratinocytes (HMK cell line), which were originally obtained from a healthy human gingival biopsy sample [29], were cultured in keratinocyte-serum-free medium containing human recombinant epidermal growth factor, bovine pituitary extract (17,005,075, Gibco, Paisley, Scotland), and antibiotics (100 IU/ml penicillin and 100 µg/ml streptomycin) (15,140–122, Gibco, Bethesda, Maryland, USA) at 37°C and 5% CO_2_. Culture media were replaced three times per week, and the cells were passaged weekly until when they reached an 80–90% confluence.

### Stock preparation of cyclic dinucleotides

A stock solution for each nucleotide was prepared by dissolving disodium form of cyclic dinucleotides, prepared as described by Gaffney et al. [30] in sterile water. To calculate the molarity, 2 µl of samples was heated to 45°C on a heating block for 1 h and then cooled down at room temperature for another 1 h. The UV absorption of the stock solution was measured by UV spectrophotometer at 253 nM for c-di-GMP and at 259 nM for c-di-AMP. The molarity was calculated by: molarity = absorbance/molar absorptivity of each sample. Serial dilutions with culture media were performed to reach the test concentrations (100 µM, 10 µM, and 1 µM).

### Stock preparation of P. gingivalis LPS

The stock solution (1 mg/ml) was prepared by dissolving 1 mg LPS of *P. gingivalis* (Invivogen, San Diego, USA) in 1 mL of endotoxin-free water.

### Incubation of HMK cells with cyclic dinucleotides

HMK cells (3x105/well) were incubated in 12-well plates at 37ºC and 5% CO2 for 24 h. The wells were washed with PBS three times, and fresh media with the three different test concentrations (100 μM, 10 μM, 1 μM) of c-di-GMP or c-di-AMP, either with or without *P. gingivalis* LPS (1 μg/ml), were poured. Cells that were incubated neither with LPS nor with cyclic dinucleotides were used as controls. After incubating the cells at 37°C for 24 h, growth media were collected from the wells and stored at −70°C until cytokine determinations. Cells were scraped and lysed with 300 μl of lysis buffer (50 mM Tris-Cl, 150 mM NaCl, and 1% Triton X-100). Afterwards, the lysates were collected and sonicated for 10 s. Protein levels of each cell lysate were measured with the Bradford method (Bio-Rad, Hercules, CA, USA).

### Analysis of cytokines levels

Intracellular (lysate) and extracellular (growth media) concentrations of IL-1β, IL-8, and IL-1Ra, MCP-1, and VEGF were detected by the Luminex technique (Bio-Rad, Santa Rosa, CA, USA) with the commercially optimized Bio-Plex kits (pro-human cytokine group I assays; Bio-Rad, Santa Rosa, CA, USA) according to the manufacturer’s instructions. The detection limit of the assay was 0.6 pg/mL for IL-1β, 1.0 pg/mL for IL-8, 5.5 pg/mL for IL-1Ra, 1.1 pg/mL for MCP-1, and 3.1 pg/mL for VEGF. For the data presentation, the amount of each cytokine per 1 µg of protein was calculated. All experiments were repeated in triplicate, and a set of cytokines was re-analyzed at an independent time to confirm the results.

### Analysis of ERK 1/2, JNK, and p38 protein kinases

The ERK 1/2, JNK, and p38 protein kinases were analyzed from the cell lyzates with western-blotting. Same amount of protein for each sample (0.5 µg) was mixed with 5 µl of laemmli buffer (4X) and heated at 95°C for 5 min. The samples were separated by 15% sodium dodecyl sulfate (SDS)-polyacrylamide gels and transferred to membranes (Trans-Blot® Turbo™ Transfer System, Bio-Rad, Hercules, CA, USA). The membranes were incubated overnight with primary ERK1+ERK2 Antibody (1:500 dilution, Thermo Fisher, Rockford, USA), p38 MAPK alpha Polyclonal Antibody (1:500 dilution, Thermo Fisher, Rockford, USA), JNK Pan Specific Antibody (1:1000 dilution, R&D Systems a biotechne brand Minneapolis, MN, USA), Phospho-ERK1/ERK2 (Thr185,Tyr187) Polyclonal Antibody (1:1000 dilution, Thermo Fisher, Bengaluru, India), Phospho-p38 MAPK alpha (Thr180, Tyr182) Polyclonal Antibody ((1:1000 dilution, Thermo Fisher, Rockford, USA), Phospho-JNK (T183, Y185) Antibody (1:1000 dilution, R&D Systems a biotechne brand Minneapolis, MN, USA), and β-actin antibody (1:10,000 dilution, Thermo Fisher, Waltham, MA, USA). A goat anti-rabbit IgG (H + L) horseradish peroxidase (HRP) conjugate (1:1000, and 1:2000 dilution, Life Technologies Corporation, CA, USA) and goat anti-mouse IgG (H + L) secondary antibody, HRP conjugate (1:4000 dilution, Thermo Fisher, Rockford, USA) were used as secondary antibodies. The detection of HRP was performed by the Novex® ECL Chemiluminescent Substrate Reagent Kit (Invitrogen, Carlsbad, CA, USA). The ChemiDoc™ MP Imaging System (Bio-Rad, Hercules, CA, USA) was used to detect the bands on the membranes. All experiments were repeated at least two independent times in triplicate.

### Statistical analysis

For statistical analyses, the IBM SPSS software (version 23, IBM, Armonk, New York, USA) was used. The results are expressed as the values of means and standard deviations, and one-way analysis of variance (ANOVA) followed by Bonferroni correction was used to analyze inter-group differences of cytokines levels. Kruskal-Wallis followed by Mann-Whitney U post-hoc test was used to analyze inter-group differences of ERK 1/2, JNK, and p38 protein kinases. *p*-Values <0.05 were considered as statistically significant.

## Results

**Figure 1.**
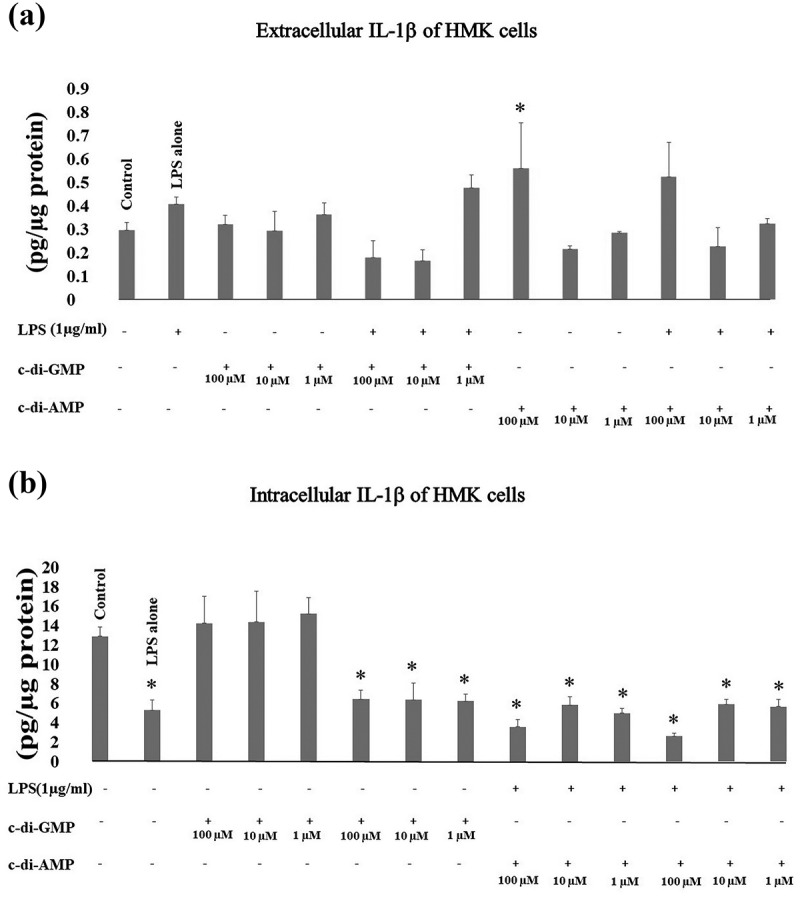
Extracellular (a) and intracellular (b) levels of IL-1β after incubating human gingival keratinocytes (HMK) with three test concentrations of c-di-GMP and c-di-AMP either alone or together with *P. gingivalis* LPS. Bars express the mean ±standard deviation for triplicate tests. * indicates a statistical difference with the control (no LPS, c-di-GMP, or c-di-AMP).

Intracellular levels of IL-1β decreased significantly when HMK cells were incubated with LPS alone (*p* < 0.001), c-di-AMP alone (at 100 µM *p* < 0.001, at 10 µM *p* = 0.001, and at 1 µM *p* < 0.001), c-di-GMP together with LPS (at 100 µM *p* < 0.001, at 10 µM *p* = 0.001, and at 1 µM *p* < 0.001), or c-di-AMP together with LPS (at 100 µM *p* < 0.001, at 10 µM *p* < 0.001, and at 1 µM *p* < 0.001). Extracellular levels of IL-1β showed a significant increase only in the presence of 100 µM of c-di-AMP alone (*p* = 0.033) (Figure 1(a,b)).

**Figure 2.**
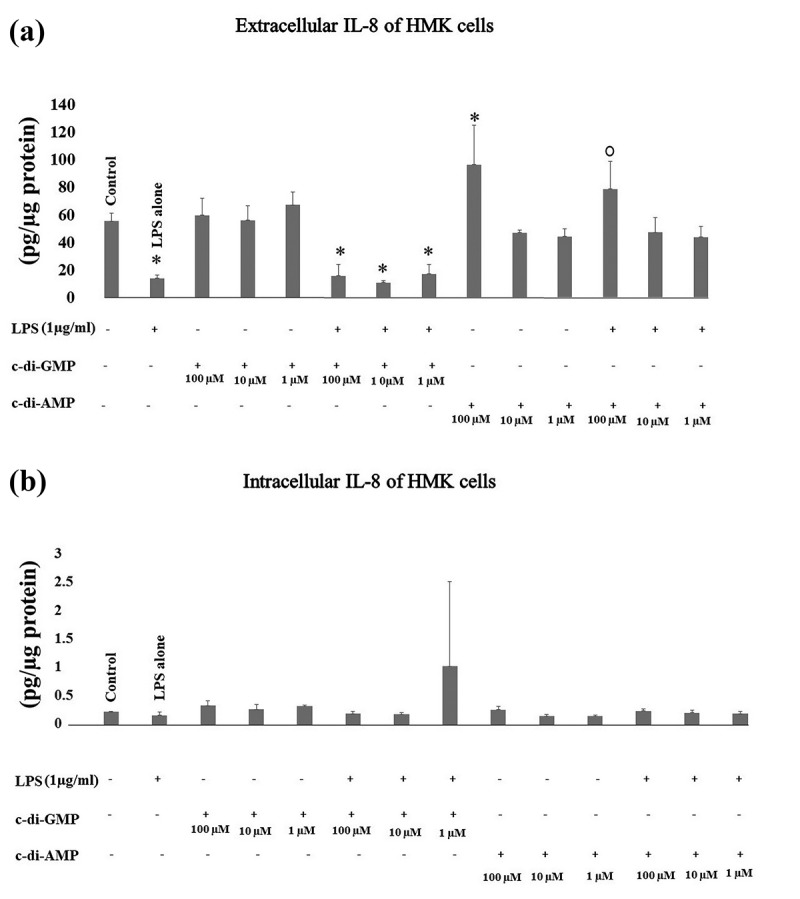
Extracellular (a) and intracellular (b) levels of IL-8 after incubating human gingival keratinocytes (HMK) with three test concentrations of c-di-GMP and c-di-AMP either alone or together with *P. gingivalis* LPS. Bars express the mean ±standard deviation for triplicate tests. * indicates a statistical difference with the control (no LPS, c-di-GMP, or c-di-AMP) and ○ indicates a statistical difference with *P. gingivalis* LPS alone.

Extracellular IL-8 levels were suppressed significantly by incubating HMK cells with LPS alone (*p* = 0.015) or LPS with 100 µM (*p* = 0.024), 10 µM (*p* = 0.006), and 1 µM of c-di-GMP (*p* = 0.037). Instead, the incubation of HMK cells with c-di-AMP in the presence of LPS neutralized the suppressive effect of LPS and kept the extracellular IL-8 levels similar to those in the control group, whereas 100 µM of c-di-AMP alone (*p* = 0.017) elevated these levels. No significant changes were observed in intracellular levels of IL-8 when HMK cells were incubated with LPS, c-di-GMP, or c-di-AMP (Figure 2(a,b)).

**Figure 3.**
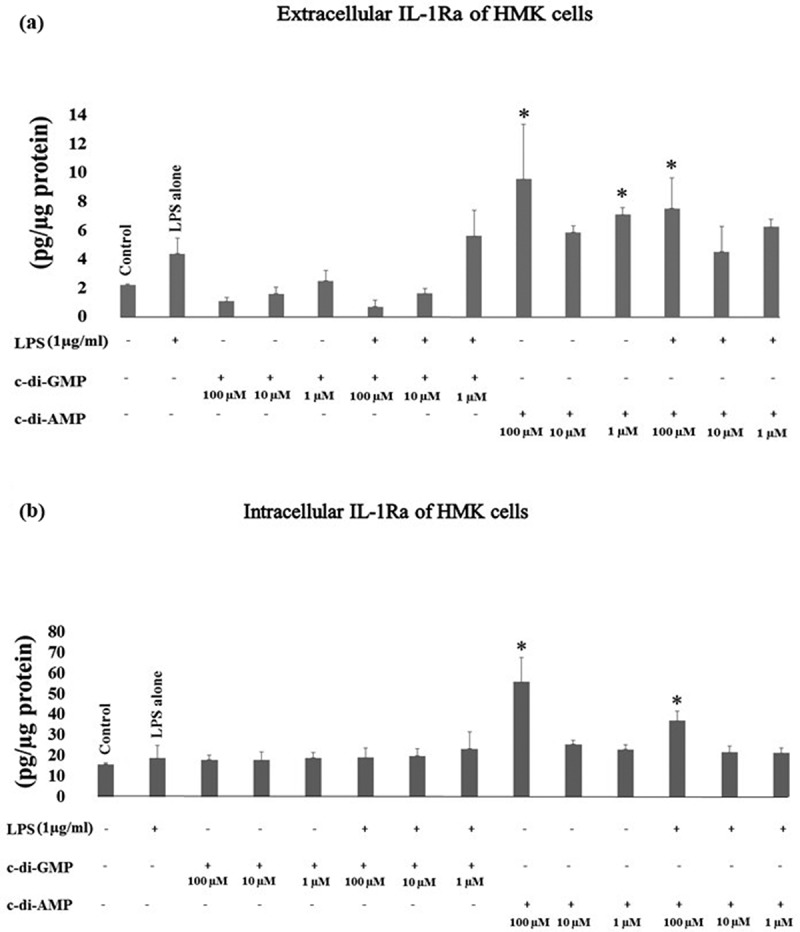
Extracellular (a) and intracellular (b) levels of IL-1Ra after incubating human gingival keratinocytes (HMK) with three test concentrations of c-di-GMP and c-di-AMP either alone or together with *P. gingivalis* LPS. Bars express the mean ±standard deviation for triplicate tests. * indicates a statistical difference with the control (no LPS, c-di-GMP, or c-di-AMP) and ○ indicates a statistical difference with *P. gingivalis* LPS alone.

Incubation of HMK cells with c-di-AMP alone (at 100 µM *p* < 0.001 and at 1 µM *p* = 0.020) or with 100 µM of c-di-AMP together with LPS (*p* = 0.007) enhanced the extracellular levels of IL-1Ra. c-di-AMP at 100 µM alone (*p* < 0.001), or with LPS (*p* = 0.002) increased intracellular levels of IL-1Ra (Figure 3(a,b)).

**Figure 4.**
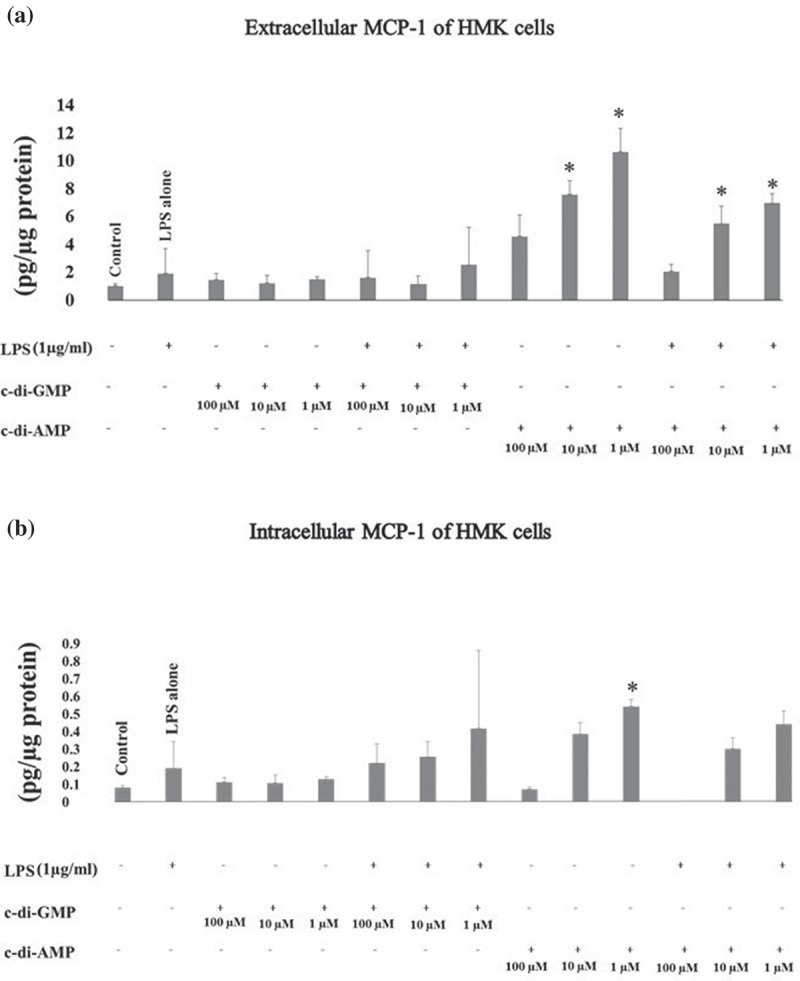
Extracellular (a) and intracellular (b) levels of MCP-1 after incubating human gingival keratinocytes (HMK) with three test concentrations of c-di-GMP and c-di-AMP either alone or together with *P. gingivalis* LPS. Bars express the mean ±standard deviation for triplicate tests. * indicates a statistical difference with the control (no LPS, c-di-GMP, or c-di-AMP) and ○ indicates a statistical difference with *P. gingivalis* LPS alone.

Extracellular MCP-1 levels were significantly elevated when HMK cells were incubated with c-di-AMP alone (at 10 µM *p* < 0.001, at 1 µM *p* < 0.001) or with LPS (at 10 µM *p* = 0.022, at 1 µM *p* < 0.001), and c-di-AMP at 1 µM increased intracellular MCP-1 levels as well (*p* = 0.025) (Figure 4(a,b)).

**Figure 5.**
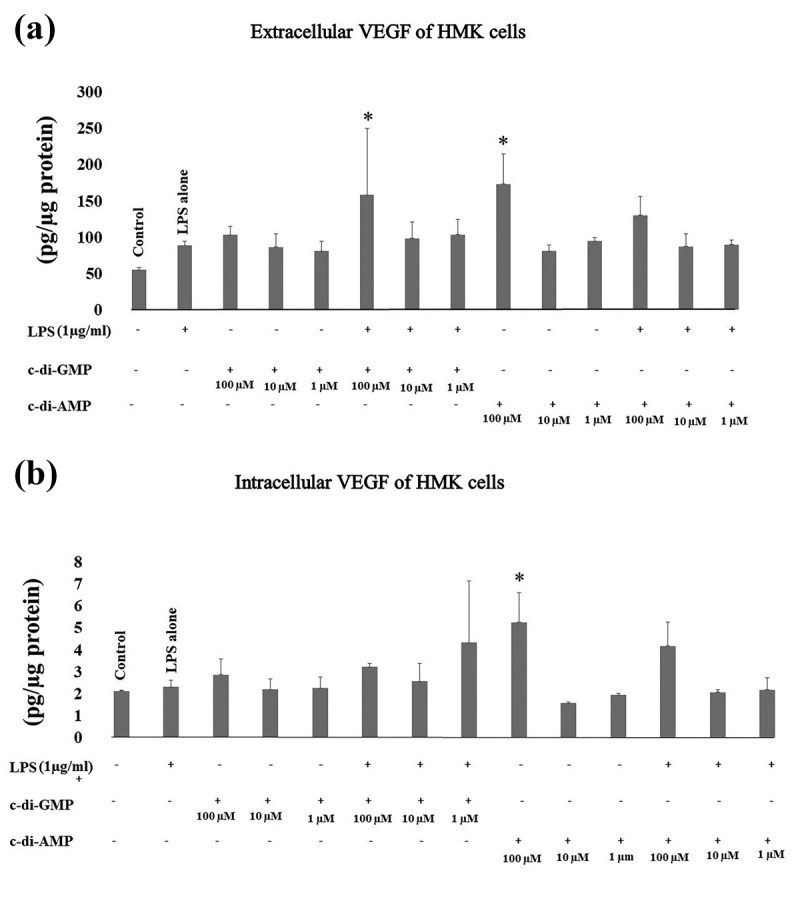
Extracellular (a) and intracellular (b) levels of VEGF after incubating human gingival keratinocytes (HMK) with three test concentrations of c-di-GMP and c-di-AMP either alone or together with *P. gingivalis* LPS. Bars express the mean ±standard deviation for triplicate tests. * indicates a statistical difference with the control (no LPS, c-di-GMP, or c-di-AMP).

Extracellular VEGF levels increased significantly when HMK cells were incubated together with 100 µM of c-di-GMP and LPS (*p* = 0.024), or with 100 µM of c-di-AMP alone (*p* = 0.005). Intracellular levels of VEGF showed a significant increase in the presence of 100 µM of c-di-AMP alone (*p* = 0.039) (Figure 5(a,b)).

**Figure 6.**
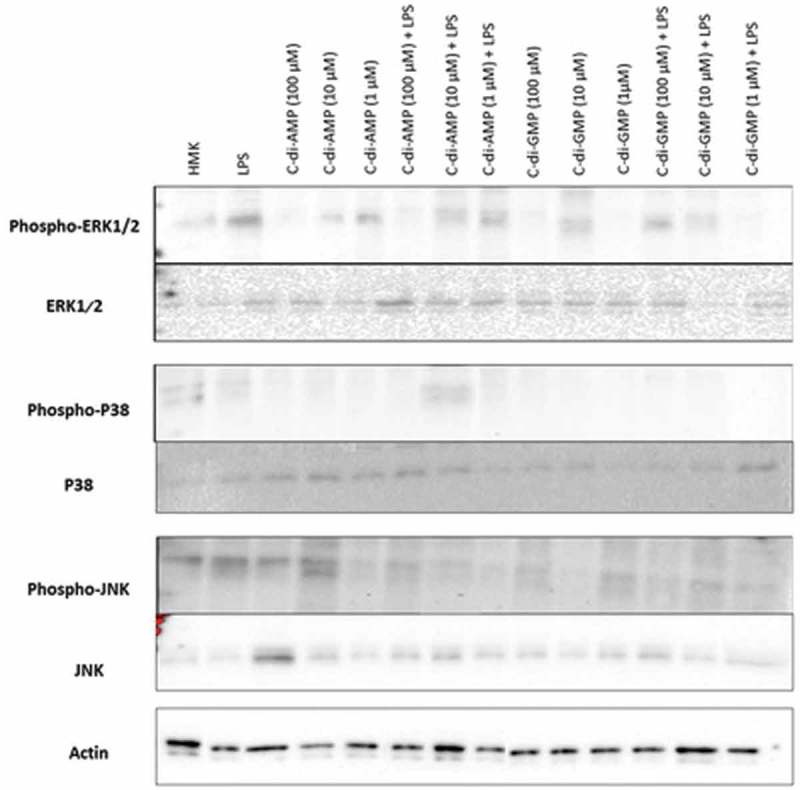
Detection of phosphorylated and non-phosphorylated forms of ERK 1/2, JNK, and p38 protein kinases pathways from cultured human gingival keratinocytes (HMK) with three test concentrations of c-di-GMP and c-di-AMP either alone or together with *P. gingivalis* LPS.

Incubation of HMK cells with *P. gingivalis* LPS resulted in an increased Phospho-ERK1/2 and a marked decrease in Phospho-p38, whereas ERK1/2, p38, JNK, and Phospho-JNK pathways did not show any response to LPS. The highest concentration of c-di-AMP (100 uM) promoted the phosphorylation of p38 and JNK kinases, whereas lower concentrations of c-di-AMP and c-di-GMP inhibited p38 phosphorylation. Moreover, c-di-GMP with LPS suppressed the phosphorylations of ERK1/2, JNK, and p38. The latter pathway, phosphorylation of p38 was down-regulated by most concentrations of both c-di-AMP and c-di-GMP alone or together with LPS (Figure 6).

## Discussion

Here, we demonstrated that c-di-AMP enhances MCP-1 and IL-1Ra and suppresses IL-1β, as well as neutralizes the LPS-inhibited IL-8 expression. To our knowledge, this is the first study to demonstrate immune regulatory effects of cyclic dinucleotides on human gingival keratinocytes. Although production of these secondary signaling molecules by bacteria and their role in bacterial behavior have been investigated [4,9,31], the effects of cyclic dinucleotides on different types of eukaryotic cells remain poorly characterized.

LPS is an outer component of Gram-negative bacterial membrane, which modulates the innate host response [32]. Our results showed that extracellular levels of IL-8 and intracellular levels of IL-1β decreased significantly after incubation of HMK cells with *P. gingivalis* LPS. LPS had no effect on the expression of other cytokines tested. According to previous studies, LPS can activate monocytes and macrophages after it interacts with circulating LPS-binding protein (LBP) [33,34]. *P. gingivalis* LPS down-regulates LBP-induced IL-8 mRNAs in human oral keratinocytes [35], whereas LPS from *Aggregatibacter actinomycetemcomitans* induces IL-1 mRNA expression and its protein synthesis in human gingival fibroblasts [36].

In the present study, it was demonstrated that c-di-AMP enhances both extracellular and intracellular levels of IL-Ra, but suppresses intracellular IL-1β levels. Moreover, c-di-AMP induced a five-fold increase in extracellular MCP-1 levels. It has a stimulating activity in host immune response and can be recognized by an endoplasmic reticulum membrane adaptor, which activates nuclear factor kappa-light-chain-enhancer of activated B cells (NF-κB) to induce the production of proinflammatory cytokines [37,38]. According to our results, c-di-AMP stimulates extracellular levels of IL-8 and VEGF only at its highest concentration, indicating that elevated levels of c-di-AMP messenger molecules of Gram-positive bacteria may have a role in vascularization and neutrophil chemotaxis. One interesting finding of this study was that c-di-AMP neutralized the suppressive effect of LPS on IL-8 expression. No similar action was observed when LPS and c-di-GMP were incubated together.

According to our results, the lowest concentration of c-di-AMP elevated the extracellular levels of MCP-1, while this effect disappeared with the increase in c-di-AMP concentration. The observation is in concordance with the concept of hormesis, a phenomenon of a dose-response concept of pharmacological active substances that are characterized by a low-dose stimulation and a high-dose inhibition [39]. The effect on monocytes has been demonstrated previously; the exposure to bacterial LPS and different PAMPs at low doses led to increased tumor necrosis factor-α and IL-6 levels, whereas high concentrations induced immune tolerance represented by a decreased release of these cytokines [40].

In our study, the incubation of human gingival keratinocyte cells with c-di-GMP did not show any significant effect on extracellular and intracellular levels of IL-8, MCP-1, VEGF, IL-1β, and IL-1Ra. Cyclic dinucleotides can stimulate the innate immune response by triggering the STING pathway [41–43]. This binding recruits and activates the TANK-binding kinase 1 (TBK1) and IκB kinase-related kinase (IKK), and elicits an innate immune response [44–46]. Activated TBK1 and IKK phosphorylate NF-κB and interferon regulatory factors (IRF3), respectively. This, in turn, leads to the production and excretion of interferon (IFN)-α, IFN-β, and other cytokines, which regulate the immune response to invading pathogens [47]. As seen in the present study, c-di GMP reduced extracellular levels of IL-8 and intracellular levels of IL-1β only when it was used together with LPS. This indicates that the suppression of both cytokines was due to the effect of LPS rather than that of c-di GMP. It has been shown that c-di-GMP can trigger the production of IL-8, MCP-1, and IFN-β, promote the recruitment and activation of macrophages and NK cells, and enhance dendritic cell maturation [48–50]. It also can stimulate the type I interferon response and increase the secretion of cytokines and chemokines to initiate a balanced Th1/Th2 response and to stimulate the inflammasome pathway and immune cell activation [49–53]. In rodents, c-di-GMP has been found to be a strong inducer of dendritic cell maturation, cytokine and chemokine expression, monocyte activation, and granulocyte recruitment [49,54]. Since the majority of these studies used mouse models and did not test the cytokine response of human epithelial cells, the differences in the results between the current study and previous studies may be allocated due to different cell types and species tested.

In comparison to c-di-GMP, c-di-AMP is a potent activator of macrophages and dendritic cells [55]. c-di-GMP has two negatively charged phosphate groups that limit its passage through the plasma membrane, and to demonstrate its immune stimulatory properties, c-di-GMP need to be used at relatively high concentrations or be transfected to the cytosol [56,57]. The molecular differences between c-di-GMP and c-di-AMP are beyond our observational report. Future studies could shed light on the cellular receptors that induce different cytokine responses against these cyclic dinucleotides.

According to the present study, *P. gingivalis* LPS stimulates phosphorylation of ERK1/2 and inhibits phosphorylation of p38, while incubating LPS and c-di-AMP together elevates Phospho-p38 level. As the same phenomenon was observed in IL-8 levels as well, it can be argued that c-di-AMP regulates the secretion of IL-8 from HMK via the Phospho-p38 pathway. This could be due to complex regulation of p38 phosphorylation that involves different players with different affinities for c-di-AMP. Another interesting finding was that both Phospho-JNK pathway and MCP-1 levels were stimulated only by c-di-AMP, either in the presence or absence of LPS. As shown previously, MCP-1 can activate the Phospho-JNK pathway in human endothelial cells [58]. To our knowledge, there is no information on the relation between MCP-1 and Phospho-JNK in human gingival keratinocytes.

In the present study, c-di-GMP suppressed phosphorylation of p38, JNK, and ERK1/2 only when incubated together with LPS. Stimulation of HMK cells with c-di-GMP alone did not affect their cytokine secretion, however, incubating c-di-GMP together with LPS suppressed IL-1β and IL-8 levels. As the production of interleukins in keratinocytes is related to the phosphorylation of p38, JNK, and ERK1/2, it is possible to claim that c-di-GMP modulates interleukin expression by regulating the phosphorylation of MAPK pathways [59].

In conclusion, in the limits of this study, c-di-AMP and, to a lesser extent, c-di-GMP regulate keratinocyte cytokine response, either as an aggregator or as a suppressor of LPS, depending on the cytokine type.
